# Small-Diameter Blood Vessel Substitutes: Biomimetic Approaches to Improve Patency

**DOI:** 10.3390/biomimetics9020097

**Published:** 2024-02-07

**Authors:** Jean-Marc Behr, Yee Shan Wong, Subbu Venkatraman

**Affiliations:** 1 SMD Swiss Medical Devices AG, 8222 Beringen, Switzerland; 2 Biomedical Engineering, School of Engineering, Temasek Polytechnic, Singapore 529757, Singapore; yeeshan@tp.edu.sg; 3 Materials Science and Engineering, National University of Singapore, Singapore 117575, Singapore; 4 iHealthTech, National University of Singapore, Singapore 117599, Singapore

**Keywords:** blood vessel, biomimetic, electrospinning, compliance

## Abstract

Small-dimeter blood vessels (<6 mm) are required in coronary bypass and peripheral bypass surgery to circumvent blocked arteries. However, they have poor patency rates due to thrombus formation, intimal hyperplasia at the distal anastomosis, and compliance mismatch between the native artery and the graft. This review covers the state-of-the-art technologies for improving graft patency with a focus on reducing compliance mismatch between the prosthesis and the native artery. The focus of this article is on biomimetic design strategies to match the compliance over a wide pressure range.

## 1. Introduction

Small-diameter blood vessels are required in coronary bypass and peripheral bypass surgery to circumvent blocked arteries. A small-diameter artery has a diameter of less than 6 mm, and typically is about 2–4 mm. In replacing vessels of diameters greater than 6 mm, vessels made from polytetrafluoroethylene (PTFE), or polyethylene terephthalate (PET) have been successful in terms of long-term patency. The PET vessel can be either woven or knitted fabric, while the PTFE is typically an extruded, biaxially oriented tube (stretched in 2 orthogonal directions after extrusion), in both cases yielding a level of porosity. When available, the replacement vessel of choice is the autologous greater or lesser saphenous vein or radial artery [1,2,3,4,5,6].

What are the patency rates of these in large diameter and small diameter replacements? Although there is a range reported for primary patency rates (i.e., patency rates long-term without any intermediate intervention), we focus on the (prospectively) randomized clinical trials reported by Klinkert et al. [1]:

It is evident that at 5 years, the mean patency rate for saphenous vein graft (74%) is superior (statistically) to PTFE graft (39%) in small dimeter arteries (above the knee femoropopliteal, for which most data exist). What are the main reasons for the low patency rates? The following factors may contribute:

- Thrombogenicity of the graft surface
- Intimal hyperplasia at the distal anastomosis
- Compliance mismatch between the native artery and the graft

**Figure 1:**
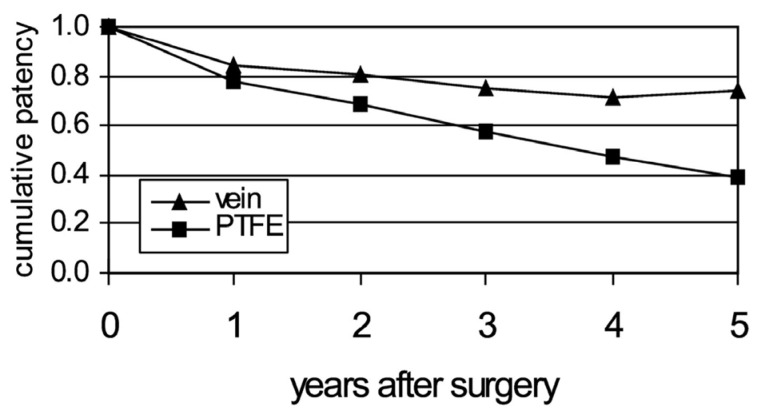
Long-term patency rates for vein and PTFE grafts above-knee femoropopliteal bypass, for six randomized clinical trials (RCT). Adapted with permission from [1] 2006, Elsevier.

Based on Figure 1, in general, all these factors result in changes in sub-endothelial tissue (the very lack of an endothelial cell layer on the graft surface can trigger this) leading to intimal hyperplasia of smooth muscle cells migrating from the media to the intima. Eventually, the combination of thrombus formation (if any) and the intimal hyperplasia (IH) leads to decreased patency and the need for re-intervention [1,2,7].

## 2. Approaches to Improve Graft Patency

### 2.1. Surface Modification

Several approaches to reduce thrombogenicity have been described [8]. It should be noted that all synthetic materials used in grafts are thrombogenic to varying degrees; as such, all need surface functionalization of some sort to minimize thrombus formation. Most strategies revolve around the use of (low molar mass) heparin on the lumen-facing side of the graft; the heparin molecule binds to anti-thrombin resulting in a fairly thrombo-resistant surface. Although these work for blood-contacting surfaces in the short-term (a few hours in hemodialysis, for example), their usefulness in the longer-term (months to years) remains to be demonstrated. For example, while coatings on stents (Hepacoat, a coating of heparin) prevent subacute thrombosis, the longer-term effects are unknown, and patients would still be prescribed anti-coagulant medication. In the case of vascular grafts, W. G. Gore has introduced a heparin-coated PTFE graft named the Gore Viabahn Endoprosthesis, used for hemodialysis of patients with end-stage renal failure who need frequent treatment. This reduces the re-intervention rates by 40% of patients that need an arterio-venous graft for hemodialysis. The time period for this to be effective, is not specified on the website [9] but may be assumed to be of the order of a few days.

An interesting approach was presented by Badv et al. [10] by covalently attaching silanized antibodies to an ePTFE graft. The surface of the ePTFE was subjected to high pressure oxygen plasma treatment, which introduced hydroxyl groups that allowed the formation of a covalent bond to the silanized anti-CD34 antibody. This approach had the advantage of requiring only two steps to functionalize the surface and in addition avoided the use of coupling agents. These surface modified ePTFE vascular prostheses showed characteristics of promoting endothelialization, while it hindered the attachment of any undesired proteins, cells or bacteria, thus showing improved resistance to blood clot formation. It should be noted that such functionalization does alter the mechanical behavior of the construct.

### 2.2. Tissue-Engineered Vascular Grafts (TEVGs)

The first of the biomimetic approaches to thrombus reduction was the use of decellularized matrices incorporating (mainly) collagen and elastin, the two main proteins found in the intimal layers of blood vessels. A few have been approved for use in Arterio Venous Fistulas (AVF), including LeMaitre’s Artegraft, Solco Basle’s Solcograft and Solcograft-P, derived from bovine artery/ureter, CryoLife’s Synergraft model 100, derived from cadaver artery (CryoLife is now Artivion), and decellularized bovine mesenteric vein (Hancock Jaffa Labs’ ProCol and Labcor’s L-Hydra graft). These acellular products have been evaluated in several animal studies [11,12,13], and are not found to be superior to PTFE grafts in terms of patency, although infection rates are reportedly lower [14]. In the case of Synergraft in particular, it is claimed that the immune system attack on the graft is reduced compared to “standard” cryo-preserved pulmonary allografts, which presumably carry the leukocyte antigen. Some of these products are now used in larger-diameter vessels such as the aorta, but there is no reported continued use in small-diameter vessels [15].

To summarize the situation with regards to the first biomimetic approach to vascular grafts:

- Although thrombogenicity may be reduced compared to PTFE grafts, the patency rates are still as low as that of PTFE grafts in small-diameter arteries.
- Therefore, the other two causes of patency loss of restenosis, namely NIH caused by anastomoses and by compliance mismatch, may be responsible for the poor performance of decellularized vessels used as vascular grafts.
- Cellularization may resolve some of these issues leading to better patency for such grafts. Cellularization may be performed in situ after implantation by decorating the lumen surface of the graft with RGD motifs or integrin-binding domains. This mandates cellular access with pores in the graft that are sufficiently large, but not large enough to cause blood leakage. That has been very hard to fabricate and control, hence endothelization in situ has been poor [2,6,16,17,18,19,20].

To overcome this, grafts have been pre-seeded with endothelial cells (autologous) or with precursor cells. This takes us to the realm of tissue engineered vascular grafts (TEVGs). The preferred substrate for all TEVGs to date has been a decellularized vascular matrix. The use of a decellularized vascular matrix in principle accomplishes two things:

- The presentation of a favorable substrate made of collagen and elastin that enables cell seeding and biochemical modification of the attached cell phenotype to the desired phenotypes (endothelial/smooth muscle cells etc.).
- The mechanical characteristics of the substrate are closer to those of native vessels, although a truly biomimetic matrix will have design features similar to that found in the native artery, as will be explained in later sections [6].

The progress in TEVGs has been well-covered in a 2016 review [15]. We will summarize the main points pertaining to the status of TEVGs as alternate materials.

- In spite of the promise afforded by a tissue-engineered blood vessel substitute, a TEVG product is yet to be approved, although one or two are already in clinical use.
- The stability of the adhered cell constructs, and the long time involved in static culturing and cell penetration into the matrix, are both stumbling blocks that hinder the development of an off-the-shelf graft available for emergency procedures.
- Regulatory aspects of the product (this is a device/biologic combination product) are complex and may require several design verification tests of the product.
- Cell choice/selection remains a heavily researched aspect of TEVGs: stem cells vs precursor cells vs a combination of endothelial cells and SMCs: autologous vs allogeneic vs xenogeneic.
- Regardless of the choice of cells, even a fully cellularized matrix vessel remains far from the ideal substitute upon implantation; almost none approaches the highly non-linear compliance behavior of native arteries.

One TEVG that has been in clinical use was developed at the Heart Institute of Japan [21,22] and implanted in pediatric patients (median age of 5.3 years). The graft was 12–24 mm in diameter and was made of a sheath of Poly Glycolic Acid (PGA) and a porous inner layer of a copolymer of poly-ε-caprolactone (PCL) and poly-L-lactide (PLA) (50:50); autologous bone marrow cells were aspirated and seeded onto the scaffold. Medium-term (~490 days) and long-term (5.8 years) results were reported, with no reports of major adverse effects such as aneurysm formation or full stenosis; partial stenosis, when observed, was resolved through angioplasty. It must be noted that this was not an off-the-shelf product, cell seeding was performed while the patient was prepared for surgery, and the diameter was fairly large. Nevertheless, it is encouraging that the patients (with congenital heart disease) survived and reportedly are doing well.

**Figure 2:**
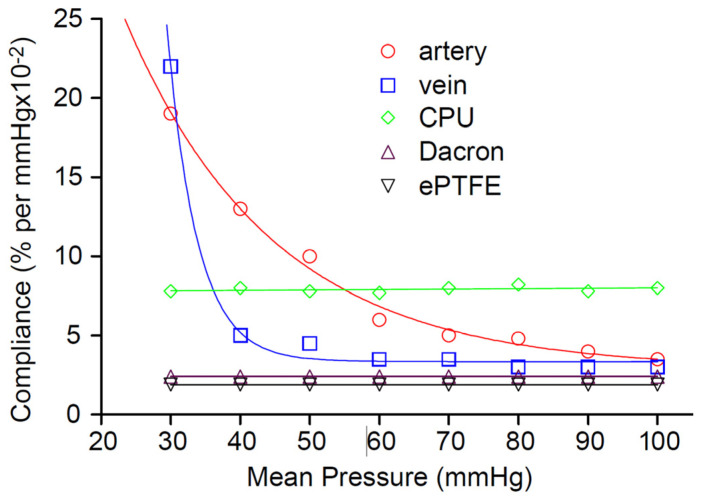
Change of compliance over luminal mean pressure. Dacron (PET), ePTFE and CPU do not exhibit the non-linear nature of veins and arteries. The latter show a change of compliance upon increase in luminal pressure [23,24]. Reprinted with permission from [23].

In spite of the advances in scaffolds for TEVGs, the implanted graft seldom matches the non-linear complex behavior of the native artery. This behavior is depicted below in Figure 2:

As it can be seen in Figure 2, both arteries and veins exhibit a compliance profile, where the compliance decreases as pressure increases; this is precisely the behavior needed to accommodate pulsatile blood flow. At low pressures, elastin fibers (elastic modulus: ~0.6–1 MPa) bear the initial strain, and compliance should be high. However, as pressure increases, the conduit should turn increasingly stiff, and this is accomplished by an increase in modulus brought about by macroscopic straightening of coiled collagen fibrils (elastic modulus around ~1 GPa) surrounding an elastin core. Without this non-linearity, vessels would develop aneurysms at higher pressures, as it has been observed with some decellularized matrices in animal testing [25,26].

### 2.3. Design Strategies to Reduce Compliance Mismatch

One way to find a truer biomimetic match would be to *mimic the design and construct of the elastin contiguous tube, surrounded by highly disordered collagen-like fibrils*. Several approaches to accomplish this have been outlined by one of the authors in his PhD thesis [17].

In order to match the biomechanical properties of arteries, elastomeric materials such as ePTFE, PET, PCL, poly-(L-lactide-*co*-caprolactone) (PLC), poly (glycerol sebacate) (PGS) and polyurethane are widely used for the construction of elastin-like contiguous tubes. With the advance of biomimicry, tropoelastin and elastin-like recombinamers (ELRs) are also being explored. One advantage of utilizing biodegradable materials as the construct is that they allow the infiltration of cells as the materials degrades over time. While for the mimicking of the collagen behavior at high strain, stiffer materials such as poly (L-lactic acid) (PLLA), PCL, silk fibroin and collagen fibril are used, together with the design of a mechanical reinforcement layer to provide the increase in modulus at high pressure. Early attempts to mimic this design with a non-cellular scaffold was reported in *Nature Medicine* in 2012 [27]. This study evaluated a rapidly degrading elastomer known as PGS that was made into a porous tubular structure and sheathed with a non-permeable PCL mesh. The sheath enables a leak-free construct, while the porous PGS allows cellular penetration while retaining static compliance values close to that of native arteries. This combination, when implanted as an aortic graft in mice, showed full cellular ingrowth and coverage by day 90. The resulting conduit at day 90 showed the non-linear compliance behavior typical of arteries, thus approaching the ideal construct in situ rather than ex vivo. It should be noted that the cellularization and subsequent deposition of elastin and collagen in the intra-cellular space happened in situ; during the early period following implantation, compliance mismatch existed.

From further survey of the literature, there appears to be no single-material candidate that can mimic the non-linear mechanical property profile of arteries. This has been recognized by various research groups, which led them to fabricate composite vessels in order to achieve a natural arterial mechanical behavior. Bezuidenhout et al. [28] for example developed a vascular prosthesis consisting of a polymeric tube with an outer fabric sock. The chain-like structure of the sock displays a non-linear elastic stress–strain response (J-Curve), which interacts with the polymeric tube in a way that it creates an arterial-like mechanical behavior. Furthermore, the porous structure of the polymer tube enables tissue ingrowth. Finite element method modelling was carried out on this system as well as in vitro static and dynamic tests, however, no in vivo tests have been reported for this composite tube [29,30].

**Figure 3:**
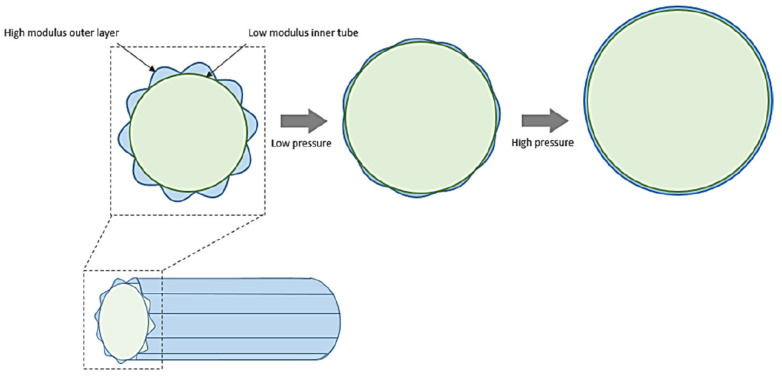
Schematic of the bilayered construct of Chen et al. [31] with the soft inner layer and the stiffer crimped outer layer that may expand upon luminal pressure increase [33]. Reprinted with permission from [33].

Another approach by Chen et al. [31] followed the same principle of combining a soft inner with a stiffer outer material. This design consists of a soft inner lining and a stiff outer layer with a crimped structure, as shown in Figure 3 [31]. At low inner pressures, the soft inner layer could initially expand easily but later got increasingly restricted due to the straightening of the outer layer. Tests have shown that at low luminal pressures the compliance has been high, whereas above pressures of higher 80 mm Hg, the compliance reduced. PET prostheses were used as a control group. At low pressures, the compliance of the composite tube was higher, while the PET prostheses were then more compliant at elevated pressure. However, native arteries were overall more compliant than these composite tubes. The design has potential but would require further optimization to better match the mechanical properties of natural arteries. Certainly, this composite design produces a non-linear elastic behavior, but Singh et al. [32] found such a bi-layered structure may be prone to kinking, which would reduce its potential for clinical applications. 

**Figure 4:**
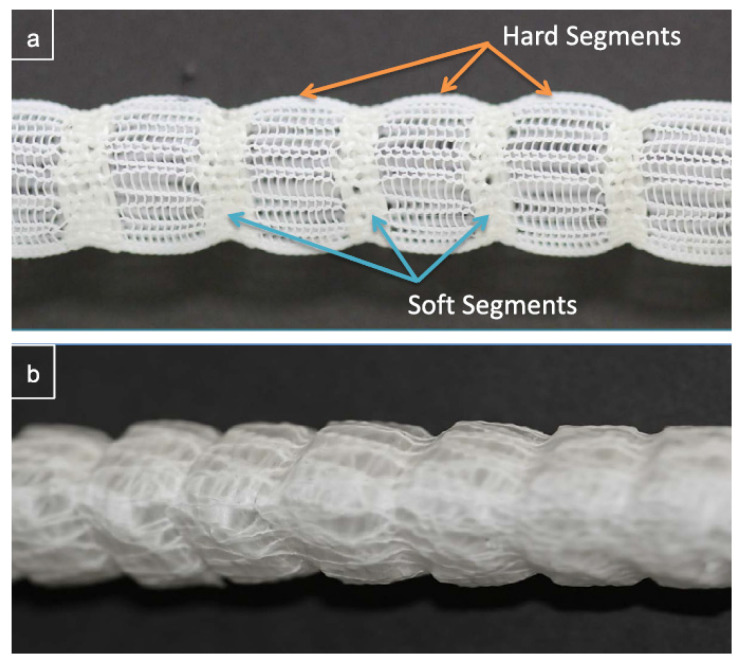
Cat-SG grafts presented by Singh et al. [33]. (**a**) Uncovered mesh with the soft (blue) and hard (orange) segments. (**b**) Cat-SG graft with the mesh being covered by PU. Reprinted with permission from [33]. 2014, Elsevier.

Singh et al. [33] published a study that involved a polyurethane (PU) covered caterpillar-inspired stent-graft (Cat-SG), which is based on a knitted structure. Figure 4 shows a schematic of their prosthesis with marked soft and hard segments. The soft segment does not only allow radial expansion at low modulus, but also contraction and extension. Easy expansion of the soft segments is given, until their diameter starts to match the one of the hard segments. This produces an arterial-like behavior showing a transition from high to low compliance upon increasing luminal pressure. Besides the mimicking of arterial compliance, these composite tubes had the advantage of being quite resistant to kinking. Up to this day, no in vivo studies have yet been carried out for these Cat-SG tubes.

**Figure 5:**
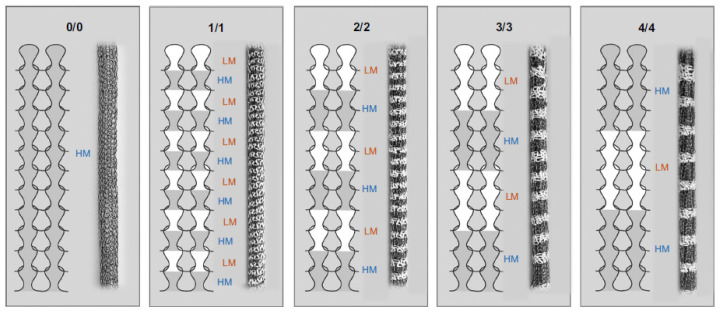
Segmented meshes of the vein graft developed by Singh et al. [34]. The mesh consists of knitted segments of soft/low modulus (LM, white) and hard/high modulus (HM, grey). The meshes provide external support to create an arterial-like compliance behavior. Reprinted with permission from [34]. 2015, Elsevier.

The same author published a study about a similar structure only one year after reporting the Cat-SG graft vascular prostheses. The outer structure consisted of a multi-segmented mesh of hard (Nitinol and polyester) and soft regions (PU). The study presented four different mesh types that differed in lengths of its soft and hard segments (Figure 5). A silicon balloon was inserted into the mesh grafts for compliance testing. All four mesh types displayed a non-linear elastic behavior, i.e., initially high compliance, followed by a reduction upon pressure increase. The grafts with long segments (4/4 samples) showed a more noticeable decrease in compliance [34]. The meshes were found to be flexible during bending, which indicates kink resistance being given. These external mesh-supported vein grafts showed promising results, however, animal testing has not been reported since this publication.

Zhang et al. [35] created a three-layered vascular graft that included a woven fabric layer of spandex yarns. The other two layers consisted of Pellethane^®^ thermoplastic polyurethanes 5863-82A, whereas the inner layer was formed by wet-spun fibers and the outer layer through spray coating. Five different tubes were fabricated, which only differed in the fineness of the spandex yarn. The fineness of the spandex yarn had an impact on the dynamic compliance of the tubes. In contrast to Bezuidenhout [28] and Singh et al. [34] that had similar concepts, the designs of Zhang et al. did not exhibit any non-linear elasticity.

The use of elastin-like recombinamers (ELR) for the fabrication of vascular prostheses was reported by Fernandez-Colino et al. [36]. The ERLs (VKVx24 and HRGD6) were used for the fabrication of a macroporous structure that should allow tissue ingrowth. This structure was further reinforced by a textile and an electrospun PCL layer. Four types of vascular prostheses were fabricated that differed in the thickness of the electrospun PCL layer. Compliance tests revealed some degree of non-linear elasticity, showing a decrease towards elevated luminal pressures. The compliance results were compared to earlier literature values, finding that the group with a PCL layer thickness of 30 µm was similar to human arteries. The burst pressure for this group was around 700 mm Hg, which is well below the one of saphenous veins (2250 mm Hg) [36] and the internal mammary artery (5000 mm Hg) [37]. This underlines the difficulty in matching the compliance to the one of native arteries, while not compromising on other important properties like burst resistance.

Our team took a different biomimetic approach: approximate the design of the artery, more specifically the orientation of collagen fibrils around a tubular elastomeric construct. The first part of this approach was reported in 2020 [38]. This work served to identify the elastomeric biodegradable polymer as the main constituent of a cast tubular structure: this was a 70:30 copolymer of ε-Caprolactone and L-Lactide (PLC). This would be surrounded by fibers of a polymer that was melt-spun onto the PLC substrate. A few designs are being evaluated and studied ex vivo for compliance matching. The results are expected to be reported in a separate publication.

### 2.4. Fabrication Techniques

**Figure 6:**
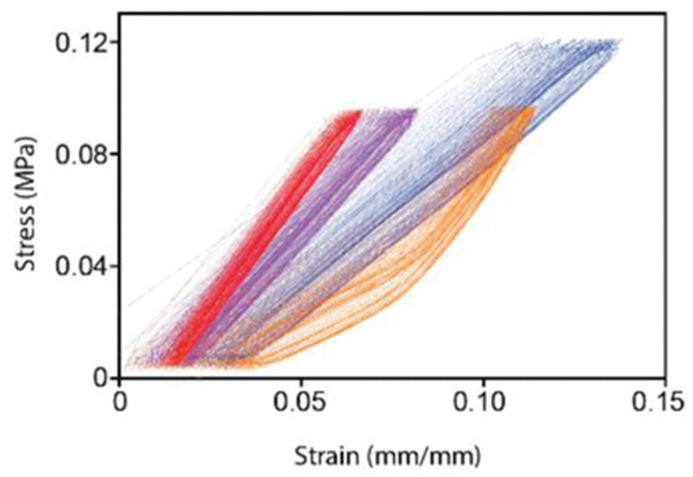
Dynamic tensile stress strain curve, with ramp up and ramp down in strain. Red data: 50% Tropoelastin (TE) blended with Poly Glycerol Sebacate (PGS); Purple: 100% TE; Blue: 70% TE; and Orange: 30% TE. Curves show minimal hysteresis, with TE30 showing the desired stress-strain behavior [40].

In the recent decade, electrospinning has been a very popular method for fabricating vascular prostheses [7,39]. There have been studies using this approach aiming to mimic the non-linear mechanical behavior of native arteries. For instance, Wang et al. [40] generated a compliance-matching construct by electrospinning a matrix of a blend of PGS with tropoelastin, which is a naturally-derived, semi-crystalline form of elastin that the authors claim mimics the elastin type found in the elastic lamellae and internal elastic lamina (IEL). This combination of materials, although electrospun, nevertheless is non-porous at the time of implantation. Of the blends studied, the one with 30% tropoelastin (TE30) appeared to mimic the non-linear mechanical behavior of the native arteries, see Figure 6. However, the authors rejected this specific composition on the basis of lack of consistency in processing. The other blends (TE50, TE70 and TE100) exhibited sufficient compliance at lower pressures to merit further consideration. Nevertheless, the implanted matrices did not fully mimic the non-linear compliance behavior of the native artery.

In mice, as an aortal segment replacement, the implanted vessel construct appears to develop circumferentially aligned smooth muscle cells as well as elastic lamellae that approximate the native lamellae. The whole process took 8 weeks, and only the TE50 (i.e., 50% Tropoelastin and 50% PGS electrospun construct) was tested in vivo. At the time of implantation this construct had no measurable pores; presumably the heat treatment of the spun fibers (160 °C for several hours) resulted in filling of the original pores by the PGS material and was therefore a good candidate construct to implant in vivo. Recall that the earlier construct [27] needed a PCL “sheath” over the electrospun PGS to ensure no leakage after in vivo implantation.

Furthermore, Niu et al. [41] proposed a vascular prosthesis of electrospun PLC 50:50 fibers. Three types of tubes were fabricated consisting of randomly aligned (RA), coaxially aligned (CA) and axially aligned (AA) fibers. Radial tensile tests as well as compliance tests were carried out. The results showed that after a pre-stretching step, all three types of vascular prostheses showed non-linear elasticity. The tensile tests were carried out in a cyclic manner, displaying clear signs of non-linear elasticity and hysteresis. The compliance tests were only carried out at physiological pressure (80–120 mm Hg). No data for the compliance at other mean pressures were recorded.

Xie et al. [42] fabricated composite vessels of a knitted PLA and electrospun PLC 50:50. For some of the samples, the knitted or electrospun layers were coated with elastin/collagen. No notable non-linear elasticity was observed for the compliance tests. The authors themselves stated that despite achieving very satisfying results regarding suture retention, circumferential tensile and burst strength, no notable improvements could be achieved regarding the compliance. 

**Figure 7:**
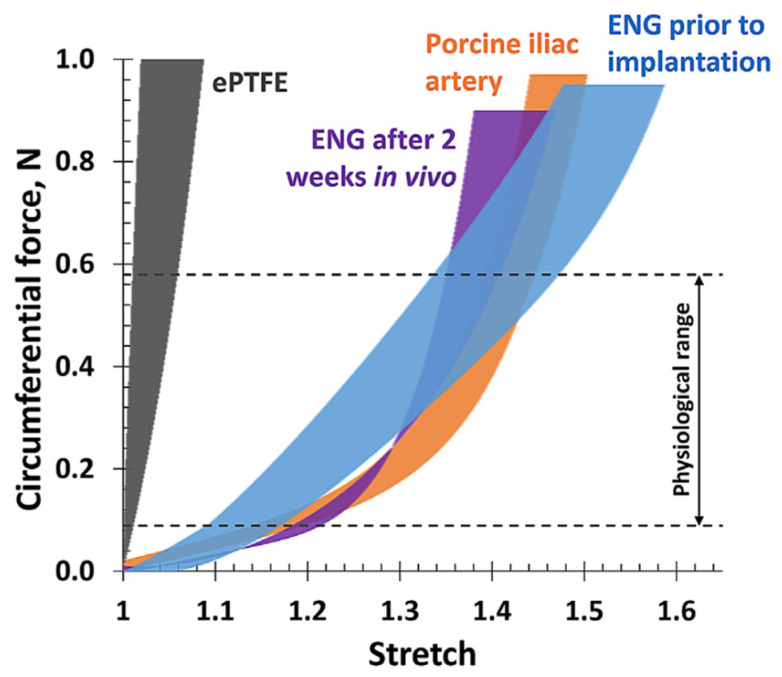
Mechanical behavior of the electrospun tubes (elastomeric nanofibrillar grafts, ENG) developed by Malecki et al. [43] showing a clearly non-linear behavior pre- and post-animal testing. The tensile properties of the grafts post-animal testing showed an even closer match to the porcine iliac arteries compared to before. Reprinted with permission from [43]. 2021, Elsevier.

A two-material electrospun vascular graft was proposed by Maleckis et al. [43]. The electrospun tubes consisted of two types of Pellethane^®^ thermoplastic polyurethanes (5863-82A and A2363-55DE). The key to their technology lays in the different shrinking degrees of the two polymers after removing the electrospun sheets from the substrate. The different phases restricted the relaxation of the nanofibers, which created residual stresses that produced the non-linear behavior of the electrospun tubes. The circumferential tensile tests could demonstrate the non-linearity of these tubes. Furthermore, the electrospun tubes were implanted into swine for two weeks and still exhibited non-linear elastic behavior when being subjected to circumferential tensile testing. The tensile properties post-animal testing were better matching the ones of the porcine iliac arteries (Figure 7).

The use of braided structures has the potential for mimicking the wavy collagen fibers of the adventitia due to their helical arrangement. The potential advantage of this technique is that such structures are easily radially expandable and compressible, with low bending stiffness and the ability of the fibers to recover after deformation.

A three-layered vascular prosthesis was presented by Zhang et al. [44] that consisted of electrospun sheets and braided silk-fibroin fibers. The two inner layers were fabricated through co-electrospinning PLC and silk-fibroin, each layer at different mixture ratios. The three-layered tubes displayed non-linear elastic behavior at three different pressure ranges (50–90, 80–120 and 110–150 mm Hg) during dynamic compliance testing. The burst pressures of these constructs were around ~2.5–3.0 MPa, which translated to more than 15,000 mm Hg. The inner lumen of these tubes was heparinized through surface modification using N-(3-Dimethylaminopropyl)-N-ethylcarbodiimide (EDC)/N-Hydroxysuccinimide (NHS). This modification resulted in a significantly reduced thrombogenicity compared to the non-heparinized scaffolds. The presented vascular prostheses that were fabricated by combining electrospinning and braiding showed promising properties. 

Guan et al. [45] fabricated a composite prosthesis of a braided PET tube embedded into a Calcium alginate/polyacrylamide hydrogel through mold casting. The tubes were tested on dynamic compliance at different pressure ranges in order to detect non-linear elasticity. The pure hydrogel tubes, as well as the ones reinforced with the braided fibers, showed a reduction in compliance towards increased pressures. The presence of the braided fibers reduced the compliance overall, which was due to the reinforcement. The hydrogel of the vascular graft tubes showed a porous structure; tests showed a swelling ratio of ~100–110% and biocompatibility tests were also satisfying, demonstrating no cytotoxicity and low hemolysis of 0.9%.

Additive manufacturing/3D-printing is a fabrication technique that has gained increasing attention over recent years. This technology has several advantages:

- Automation
- Flexibility in shapes
- Ability to fabricate complex shapes
- Combination of multiple materials
- Rapid prototyping
- Ability of cell loading [46,47,48]

Several studies present vascular prostheses that were fabricated through 3D-printing/-bioprinting. Such would for example involve the fabrication of 3D printed structures/matrices that will be subjected to cell seeding, fabrication of cell-loaded constructs, etc. [49,50,51,52].

Tardalkar et al. [53] fabricated TEVGs by 3D printing or molding a tubular vessel using a bioink consisting of digested caprine blood vessels, polyvinylalcohol (PVA) and gelatin. These vascular grafts were fabricated with an inner diameter (ID) of 0.5 mm and a wall thickness of ~140–180 µm. The resulting vascular grafts showed superhydrophilic properties, absorbing water droplets and exhibiting swelling of 1–5%. The bioink printed vessels were tested on their tensile properties and were notably stronger than the abdominal rat aorta controls. Not only was the ultimate tensile load exceeding one of the controls by a factor of roughly 4, even the elongation at break was around 75% greater. After implantation in seven rats, the TEVG were later subjected to immunofluorescence and histological analysis that showed recruitment of endothelial and smooth muscle cells (via α smooth muscle actin [αSMA], vascular endothelial growth factor [VEGF] and von-Willebrand-factor [vWF]). Furthermore, SEM analysis showed cellular attachment on the surface. This study highlighted the use of bioinks and 3D-printing for the fabrication of TEVG. No compliance tests were carried out, however that would be interesting for further research. 

Another study demonstrated the use of rotary bioprinting for the production of vascular prostheses. A bioink consisting of heat-treated collagen and fibrinogen was loaded with neonatal human fibroblast cells and printed onto a rotating rod. The key to their study laid in the heat-treatment of the collagen, that enabled the fibrinogen in the blend to be printable and even allowed the ink being loaded with a high density of cells. Culturing of the bioprinted vascular grafts led to more ECM deposition that led to increased mechanical properties. The burst pressure of these tubular grafts was determined through estimation being around 1110 mm Hg, which is about half of that of saphenous veins and a roughly a fifth of that of carotid arteries. No compliance tests were carried out, instead it was estimated based on circumferential tensile tests. The compliance significantly decreased over time following ECM deposition.

Zhou et al. [54] 3D printed a hydrogel tube consisting of gelatin-methacryloyl (GelMA), gelatin and sodium alginate (SA). To stabilize the shape, the tubes were cross-linked in a two-step process. The resulting hydrogel tubes were porous, demonstrating leakage for a red ink, but not for a solution containing red blood cells. Burst pressure and compliance were estimated based on conversions of radial tensile test results. Three different tubes were analyzed for this: pure SA tubes, GelMA/SA and GelMA/Gelatin/SA tubes. The results showed an interesting trend; the pure SA tubes showed the lowest burst pressure and compliance, while the GelMA/Gelatin/SA tubes were the most resistant to burst, while also being the most compliant. In general, increases in compliance are at the cost of burst pressure and vice versa. Yet, it would be interesting seeing such a tube being tested under dynamic conditions according to ISO 7198:2016 [55], which would allow better comparison to human arteries and saphenous veins. The hydrogels were tested in vivo in rats, however, not as vascular grafts. Within one week, the SA and gelatin degraded, while only GelMA remained. During the second week, cell infiltration was observed, which was regarded as proof for the replacement of the hydrogel by autologous tissue.

## 3. Conclusions

One of the major contributors to stenosis or loss of patency is the mismatch of mechanical properties between the artificial blood vessel and the native artery. In larger diameter (>6 mm in diameter) artery replacement, this does not play a significant role, but in smaller arteries, it seems to be a crucial requisite. More specifically, the dynamic compliance behavior of the artery over pressures ranging from about 30 mm Hg to about 100 mm Hg needs to be mimicked by the vascular graft. No single material can match this behavior; ePTFE, biaxially oriented, is too stiff, whereas polyurethane does not sufficiently stiffen up at higher pressures, possibly causing aneurysms in implanted vessels [23,24].

The non-linear mechanical behavior of native arteries is a consequence of both the extra-cellular matrix materials as well as of design. The early response (lower pressures) is due to the contiguous elastin matrix in the elastic lamellae when the vessel deforms to accommodate the increased pressures. The collagen fibrils that are laid over the elastin layers, are disoriented to begin with; as the vessel expands, the collagen fibrils straighten out until they are circumferentially aligned. Beyond this point of deformation, the load is transferred to the collagen fibrils, which then are sufficiently stiff to resist further expansion/ballooning as is required for prevention of aneurysm formation [25].

Earlier attempts to match compliance with mimicking centered around decellularized matrices consisting mostly of collagen and elastin with a few other proteins thrown in. Such matrices are not particularly easy to manufacture/extract and may vary in properties depending on the source of the extra-cellular matrix proteins used (a popular choice for studies was the bovine mesenteric arterial tissue). The process may also have resulted in destroying the 3D construct of the collagen and the elastin, and perhaps also led to degradation of the collagen and elastin. In any case, none of the studied matrices (some also approved) showed performance superior to PTFE-based grafts and given the issues with QC and reproducibility of properties, such decellularized constructs are no longer preferred to Dacron/PTFE grafts. 

More recent attempts to mimic this behavior have centered around the engineering of the arterial wall tissues using a bioresorbable matrix material, either alone, or as a blend component. The scaffolds may be electrospun to fibers or may be cast as tubes. At the time of implantation, these scaffolds may be seeded or non-seeded; the construct is typically stiffer than an arterial wall, and slowly degrades as it is being populated with cellular matrices. The implanted construct presumably is porous enough to attract extramural cellularization. The time process to achieve an acceptable level of cellularization, consistent with the development of elastic lamellae and aligned smooth muscle cells, takes about 8 weeks in the case of a rapidly degrading matrix (PGS) but may be longer with slower-degrading matrices such as PLA or PCL.

Our own approach to mimicking the non-linearity of mechanical behavior is to use an initially non-porous but biodegradable and elastomeric tube that is covered by a patterned disoriented fiber pattern around the inner elastomeric tube [4]. The fiber material can be biodegradable but stiffer than the tube material. Porosity is generated in situ, leading eventually to cellular deposition and integration.

In summary, in order to achieve the biomechanical properties that mimic the viscoelastic properties of a human artery, both material selection and construct design are critical to the performance of the blood vessel prosthesis.

## Data Availability

Data sharing not applicable. No new data were created or analyzed in this study.
